# Preoperative immunotherapy with atezolizumab and tiragolumab in patients with colorectal liver metastases—the PURPLE trial

**DOI:** 10.1016/j.esmogo.2025.100226

**Published:** 2025-09-01

**Authors:** S. Kasper, E. Elez, U. Neumann, S. Lang, A.-K. Trampe, F. Salva, C. Dopazo, I. Virchow, S. Hartmann, K. Herrmann, J. Tabernero, A. Westendorf, M. Schuler, A. Schramm

**Affiliations:** 1 Department of Medical Oncology, West German Cancer Center, University Hospital Essen, a member of the imCORE network, Essen, Germany; 2 National Center for Tumor Diseases (NCT), NCT West, Essen, Germany; 3 Department of HPB Surgery and Transplants, Barcelona Hospital Campus, a member of the imCORE network, Barcelona, Spain; 4 Department of General, Visceral and Transplantation Surgery, West German Cancer Center, University Hospital Essen, a member of the imCORE network, Essen, Germany; 5 Vall d’Hebron Institute of Oncology, Medical Oncology, Vall d’Hebron Barcelona Hospital Campus, a member of the imCORE network, Barcelona, Spain; 6 Institute of Pathology, West German Cancer Center, University Hospital Essen, a member of the imCORE network, Essen, Germany; 7 Department of Nuclear Medicine, West German Cancer Center, University Hospital Essen, a member of the imCORE network, Essen, Germany; 8 Institute of Medical Microbiology, University Hospital Essen, a member of the imCORE network, Essen, Germany

**Keywords:** colorectal liver metastases, preoperative immunotherapy, atezolizumab, tiragolumab, clinical trial

## Abstract

**Background:**

In colorectal cancer (CRC), the liver is the most common site of metastasis, which is the leading cause of CRC-related mortality. Hepatic resection offers long-term survival in some patients with CRC liver metastases (CRLM), but recurrence rates remain high (50%-75% within 2 years). Preoperative immunotherapy may induce tumor regression and improve long-term surgical outcomes. The PURPLE trial evaluates the feasibility, safety, and efficacy of short-term preoperative immunotherapy with the anti-programmed death-ligand 1 (PD-L1) antibody atezolizumab and the anti-T cell immunoreceptor with Ig and ITIM domains (TIGIT) antibody tiragolumab in patients with resectable CRLM.

**Study design:**

PURPLE is an international, open-label, multicenter, randomized phase II ‘window of opportunity’ trial. Patients with resectable CRLM are randomized 2 : 1 to receive two cycles of atezolizumab (840 mg) plus tiragolumab (420 mg) before surgery (experimental arm) or immediate surgery (control arm). The primary endpoint is the percentage of patients with complete or major pathological regression of the resected metastases (tumor regression grade 1/2, Rubbia-Brandt criteria). The statistical design follows Simon’s two-stage approach with interim and final analyses comparing pathological response rates using descriptive and exploratory methods. Secondary endpoints include feasibility, safety, post-operative complications, and metabolic response by positron emission tomography. Exploratory studies characterize immune cell infiltration, tumor mutational burden, and circulating tumor DNA dynamics.

## Highlights

• The impact of preoperative combination immunotherapy on colorectal liver metastases is evaluated.
• The feasibility and safety of atezolizumab and tiragolumab before surgery are assessed.
• Biomarkers associating with treatment response are explored.

## Graphical abstract

**Figure:**
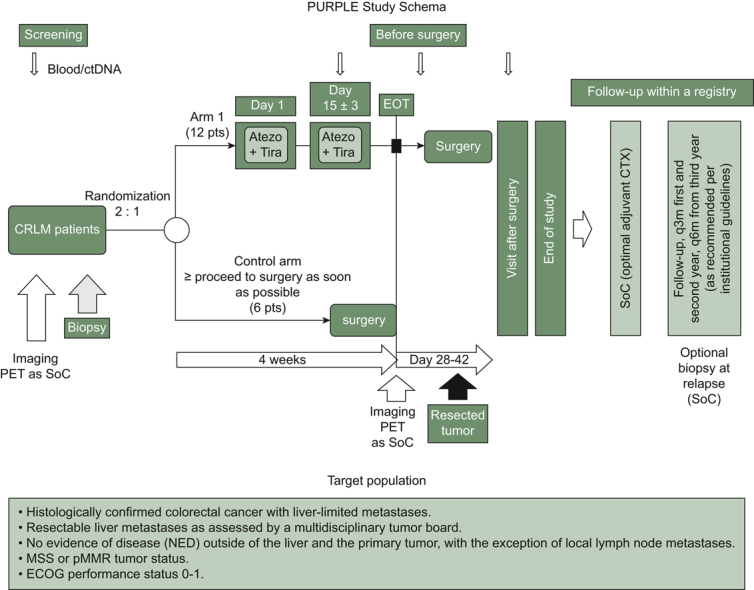

## Description of protocol

### Background

Liver is the most common site of metastases, which are the leading cause of cancer-related mortality in patients with colorectal cancer (CRC).^1^ Hepatic resection is the only treatment that can provide long-term survival for those with CRC liver metastases (CRLM), achieving 5-year survival rates of 16%-71%.^2^ However, tumor recurrence after curative resection of CRLM remains a major issue, with 50%-75% of patients experiencing recurrence within 2 years.^1^ The efficacy of immunotherapy is reduced in patients with liver metastases due to the immunosuppressive liver microenvironment, which fosters immune evasion.^3^

Immune checkpoint molecules such as programmed cell death protein 1 (PD-1)/programmed death-ligand 1 (PD-L1) and T cell immunoreceptor with Ig and ITIM domains (TIGIT) play central roles in this process: PD-L1 expression by immune cells in the resected CRLM correlates with prognosis, whereas TIGIT expression has recently emerged as a marker of T-cell dysfunction and exhaustion in several cancer types, including CRC and hepatocellular carcinoma (HCC).^4^, ^5^, ^6^, ^7^, ^8^ Mechanistically, TIGIT is expressed on activated and exhausted CD8+ and CD4+ T cells, regulatory T cells, and natural killer (NK) cells. Its ligand, CD155 (poliovirus receptor), is highly up-regulated on tumor and myeloid cells within the tumor microenvironment (TME). TIGIT binding delivers a strong inhibitory signal that potently suppresses T-cell and NK cell effector function, promotes regulatory T-cell (Treg) suppressive capacity, and favors T-cell exhaustion.^4^^,^^7^

Given that single-agent immunotherapy has shown limited efficacy in microsatellite-stable/mismatch repair-proficient (MSS/pMMR) CRC, combination strategies targeting TIGIT and PD-L1 may enhance antitumor responses by synergistically restoring T-cell and NK cell cytotoxicity and reducing Treg suppression.^9^

Clinical data from HCC, a cancer with a similar immunosuppressive hepatic microenvironment, further support TIGIT blockade: In the phase Ib/II MORPHEUS-Liver trial, addition of the anti-TIGIT antibody tiragolumab to atezolizumab plus bevacizumab led to a substantially higher objective response rate (43% versus 11%).^10^ These results provide the first clinical evidence that dual checkpoint inhibition including TIGIT in liver tumors may overcome the hepatic tumor’s intrinsic immunotherapy resistance and warrant translation into the metastatic setting.

Against this background, the PURPLE trial (Table 1) evaluates the feasibility, safety, and efficacy of a short-term preoperative immunotherapy regimen with the anti-PD-L1 antibody atezolizumab and the anti-TIGIT antibody tiragolumab in patients undergoing surgical resection of CRLM.

**Table 1:** Specifications table

**Subject area**	Immunology and Microbiology
**More specific subject area**	Colorectal cancer, liver metastases, neoadjuvant therapy
**Name of your trial in progress**	Preoperative immunotherapy with atezolizumab and tiragolumab in patients with colorectal liver metastases—the PURPLE trial
**Reagents/tools**	Atezolizumab (Tecentriq®), tiragolumab, FDG–PET–CT imaging
**Trial design**	The PURPLE trial is an international, randomized, open-label, multicenter, phase II ‘window of opportunity’ study. It assesses the feasibility, safety, and pathological tumor response of short-term preoperative immunotherapy in patients with colorectal liver metastases (CRLM). A total of 18 patients will be enrolled and randomized 2 : 1 into two arms. Patients in the experimental arm will receive two cycles of atezolizumab (840 mg) and tiragolumab (420 mg) before surgical resection, while patients in the control arm will directly proceed to surgery. The primary endpoint is the pathological regression rate in the resected metastases according to Rubbia-Brandt criteria. Secondary endpoints include feasibility, resectability, PET response, post-operative complication rates, and reasons for not undergoing surgery. Exploratory endpoints involve translational biomarker analyses, including immune cell profiling, tumor mutational burden, and circulating tumor DNA dynamics. The statistical design follows a Simon’s two-stage approach, with an interim analysis planned after six patients are treated in the experimental arm to assess feasibility. The final analysis will compare pathological response rates between the two arms using descriptive statistics and exploratory inferential methods where applicable.
**Trial registration**	EU-CT Number: 2022-503044-40
**Ethics**	The study is conducted in accordance with the Declaration of Helsinki. Informed consent will be obtained from all participants before enrollment. The trial was approved according to the Clinical Trial Regulation, CTR (EU No. 5367/2014) and the German Medicinal Products Act (AMG); part I (scientific assessment) by the Paul-Ehrlich Institute (PEI) (No. P02124) and part II (ethical assessment) by the Ethics Committee of the Julius-Maximilians University Wuerzburg
**Value of the trial in progress**	• Investigates a novel preoperative immunotherapy approach in colorectal liver metastases • Evaluates the feasibility and impact on pathological response rates • Incorporates translational research to identify potential biomarkers of response

## Study design

**Figure 1:**
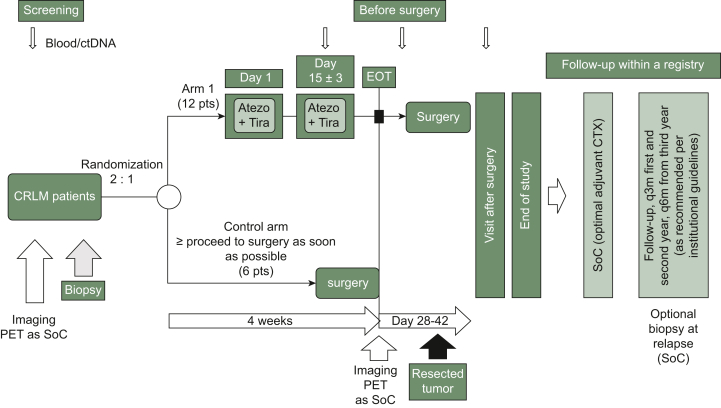
**Study design.** Atezo, atezolizumab; CLRM, colorectal liver metastases; ctDNA, circulating tumor DNA; CTX, chemotherapy; EOT, end of treatment; PET, positron emission tomography; pts, patients; q3m, every 3 months; q6m, every 6 months; SoC, standard of care; Tira, tiragolumab.

PURPLE is an international, open-label, multicenter, randomized phase II trial with a ‘window of opportunity’ design. Patients with resectable CRLM are randomized 2 : 1 to receive either two cycles (every 2 weeks) of atezolizumab (840 mg) plus tiragolumab (420 mg) before surgery (experimental arm) or direct surgery without immunotherapy (control arm) (Figure 1). The study aims to determine the feasibility, safety, and efficacy of this preoperative immunotherapy approach.

Patients in the experimental arm will receive atezolizumab and tiragolumab on days 1 and 15 before undergoing surgery at day 29 ± 3. Those in the control arm will directly proceed to surgery within the same timeframe.

A core component of the study design includes a mandatory preoperative tumor biopsy before the initiation of immunotherapy in the experimental arm to allow molecular and immune profiling. In addition, all patients will undergo [^18^F]2-fluoro-2-deoxy-D-glucose–positron emission tomography (FDG–PET) imaging before therapy initiation and again after the completion of immunotherapy before surgery to evaluate metabolic tumor response.

The primary endpoint is the percentage of resected patients with complete or major pathological regression (tumor regression grade 1 or 2 according to Rubbia-Brandt criteria). The statistical design follows Simon’s two-stage approach, with an interim analysis after 6 patients have been randomized to the experimental arm, with a total target enrollment of 18 patients (12 in the experimental arm, 6 in the control arm).

Secondary endpoints include the proportion of patients who proceed to surgery as planned, perioperative safety (graded by Clavien–Dindo classification), post-operative complication rates, and metabolic response per FDG–PET. Exploratory assessments include a comprehensive translational analysis, including immune cell infiltration, tumor mutational burden, and circulating tumor DNA (ctDNA) dynamics in response to treatment.

### Patient population and eligibility criteria

The PURPLE trial includes adult patients (≥18 years) with histologically confirmed colorectal adenocarcinoma and resectable liver metastases, as determined by a multidisciplinary tumor board. Eligible patients must have MSS or mismatch repair-proficient (pMMR) disease, an Eastern Cooperative Oncology Group (ECOG) performance status of 0 or 1, and adequate organ function.

### Inclusion criteria

• Histologically confirmed CRC with liver-limited metastases.
• Resectable liver metastases as assessed by a multidisciplinary tumor board.
• No evidence of disease outside of the liver and the primary tumor, with the exception of local lymph node metastases. If primary tumor is *in situ*, it must be resectable.
• MSS or pMMR tumor status.
• ECOG performance status 0-1.
• Adequate hematologic, renal, and hepatic function.
• Written informed consent.

### Exclusion criteria

• Presence of extrahepatic metastases.
• Prior treatment with immune checkpoint inhibitors.
• Uncontrolled infections or autoimmune diseases requiring systemic therapy.
• Previous major surgery within 4 weeks before study inclusion. Exception: surgery of primary tumor within 2 weeks before randomization is allowed.
• Any anticancer therapy, including chemotherapy or hormonal therapy or radiotherapy, within 4 weeks before randomization.
• Active or untreated central nervous system metastases.
• Pregnancy or breastfeeding.

This patient selection ensures that the trial focuses on a homogeneous population with a high likelihood of benefiting from preoperative immunotherapy while minimizing potential confounding factors.

### Study treatment

• **Experimental arm:** atezolizumab [840 mg intravenously (i.v.)] + tiragolumab (420 mg i.v.) on day 1 and day 15; surgery is scheduled at day 29 ± 3 following completion of immunotherapy.
• **Control arm:** immediate surgery or surgery after observation (up to 28 days after randomization).

### Assessments

• Tumor biopsy (before and after treatment)
• PET–computed tomography imaging before and after therapy
• Post-operative complication monitoring according to Clavien–Dindo
• Translational research (immune profiling, ctDNA clearance)

### Sample size and statistical design

The sample size calculation is based on Simon’s two-stage design. A total of 18 assessable patients will be enrolled and randomized: 12 in the experimental arm and 6 in the observational control arm. Patients must undergo liver resection within the defined timelines to be considered assessable.

A non-interesting response rate of p_0_ = 5% and a target response rate of p_1_ = 35% will be assumed. The null hypothesis (H_0_): p ≤ p_0_ versus the alternative hypothesis (H_1_): p ≥ p_1_ will be tested with a first-type error rate of α = 0.05. With 80% power and a two-sided α level of 0.05, recruitment in stage 1 will stop at six patients in the experimental arm if no response is observed. If at least one responder (complete or major pathological regression) is observed, recruitment will continue to stage 2, adding six more patients in the experimental arm (Table 2). If two or fewer responders are observed in this arm, the study will be declared negative for the primary endpoint. Assuming a 10% dropout rate, 14 patients will be enrolled in the experimental arm. Considering the control arm in addition, a total of 20 patients have to be enrolled. Additionally, an interim safety analysis will be conducted after randomization of nine patients (six to the experimental treatment arm and three to the control arm). Regarding feasibility, the study will be considered feasible if ≥80% (10 of 12) of treated patients in the experimental arm undergo surgery. Secondary and exploratory endpoints will be analyzed descriptively using means, medians, ranges, standard deviations, and confidence intervals. *P* values will be reported descriptively without multiple-testing correction.

**Table 2:** Statistical assumptions

Step	Patient recruitment (except control)	Outcome	Decision
1	*n*_1_ = 6 (12 patients in total)	0 responder	Stop recruitment, H_0_ is not rejected
		≥1 responder	Continue with step 2
2	*n*_2_ = 6 (12 patients in total)	≤2 responders	H_0_ is not rejected
		≥3 responders	H_0_ is rejected in favor of H_1_
12 assessable patients in the experimental arm			

H_0_, null hypothesis; H_1_, alternative hypothesis.

### Translational research and biomarker analysis

Recent studies have improved our understanding of immune mechanisms operative in patients receiving PD-L1/PD-1 inhibition as monotherapy. However, in patients with MSS/pMMR CRC, distinct mechanisms of immune resistance or suppression still limit the potential of PD-L1/PD-1-directed immunotherapy.

One of the key mechanisms counteracting antitumor T- and NK cell immunity and checkpoint blockade is the suppressive activity of Treg and myeloid cells. During tumor progression, different subsets of Treg and myeloid cells acquire a specific molecular program resulting in tumor-promoting and immune-suppressive activity. To decipher the effects of PD-L1 blockade and the modulation of immune cells, we have established multicolor immunofluorescence protocols together with digital image analysis to assess in great detail the spatial distribution of myeloid cells, T cells, and NK cells in distinct areas of the TME. Together with the unique pre–post comparison of metastasis samples, this will allow for in-depth analysis and insights into this type of immunotherapy.

A comprehensive translational research program will be conducted to identify potential predictive and prognostic biomarkers using immunohistochemical analyses, next-generation panel sequencing, and gene expression analysis of primary tumor samples. Particular focus will be put on the comparison of intratumoral immune cell infiltrates in the primary biopsy and the resected tumor after immune therapy, complemented by insights from an optional biopsy at relapse if available.

Further, monitoring of peripheral blood immune cells, with a focus on T cells, NK cells, and myeloid cells, will be carried out using flow cytometry.

The following parameters will be analyzed:

• Multiplexed profiling of RNA and protein expression from pretreatment tumor biopsies and resected tumors by RNA sequencing, if possible single cell RNA sequencing combined with T-cell clonality analyses.
• Phenotyping of the immune cell repertoire including, but not limited to, intratumoral and peripheral analyses of cytotoxic T cells (CD8+/GRZB+) before and after immunotherapy.
• Genomic profiling of pretreatment biopsy for determination of tumor mutational burden, human leukocyte antigen (HLA) status, potential neoantigens, and HLA loss. All parameters will be descriptively correlated with response to immunotherapy.
• Genomic profiling of pretreatment biopsy and resected tumors for determination of clonal selection upon immunotherapy.
• Number of patients with ctDNA clearance and markers of early relapse.

This immune monitoring will help to identify a signature in patients who benefit most from preoperative immunotherapy. Candidate biomarkers identified from this exploratory study should then be tested in a larger cohort for their potential to predict overall response or quality of response.

Finally, this perioperative setting is ideal for analyzing predictive biomarkers for the efficacy of combination immunotherapies in the future.

## Declaration of generative AI and AI-assisted technologies in the writing process

During the preparation of this work, the author(s) used ChatGPT v4.0 in order to improve readability and language. After using this tool, the author(s) reviewed and edited the content as needed and take(s) full responsibility for the content of the publication.
